# Autophagy suppresses the formation of hepatocyte-derived cancer-initiating ductular progenitor cells in the liver

**DOI:** 10.1126/sciadv.abf9141

**Published:** 2021-06-04

**Authors:** Valentin J. A. Barthet, Martina Brucoli, Marcus J. G. W. Ladds, Christoph Nössing, Christos Kiourtis, Alice D. Baudot, James O’Prey, Barbara Zunino, Miryam Müller, Stephanie May, Colin Nixon, Jaclyn S. Long, Thomas G. Bird, Kevin M. Ryan

**Affiliations:** 1 Cancer Research UK Beatson Institute, Garscube Estate, Switchback Road, Glasgow G61 1BD, UK.; 2 Institute of Cancer Sciences, University of Glasgow, Garscube Estate, Switchback Road, Glasgow G61 1QH, UK.; 3 MRC Centre for Inflammation Research, The Queen’s Medical Research Institute, University of Edinburgh, Edinburgh EH16 4TJ, UK.

## Abstract

Hepatocellular carcinoma (HCC) is driven by repeated rounds of inflammation, leading to fibrosis, cirrhosis, and, ultimately, cancer. A critical step in HCC formation is the transition from fibrosis to cirrhosis, which is associated with a change in the liver parenchyma called ductular reaction. Here, we report a genetically engineered mouse model of HCC driven by loss of macroautophagy and hemizygosity of phosphatase and tensin homolog, which develops HCC involving ductular reaction. We show through lineage tracing that, following loss of autophagy, mature hepatocytes dedifferentiate into biliary-like liver progenitor cells (ductular reaction), giving rise to HCC. Furthermore, this change is associated with deregulation of yes-associated protein and transcriptional coactivator with PDZ-binding motif transcription factors, and the combined, but not individual, deletion of these factors completely reverses the dedifferentiation capacity and tumorigenesis. These findings therefore increase our understanding of the cell of origin of HCC development and highlight new potential points for therapeutic intervention.

## Teaser

Autophagy represses YAP/TAZ-dependent hepatocyte dedifferentiation into liver progenitor cells to prevent tumorigenesis.

## INTRODUCTION

Liver cancer is predicted to be the third leading cause of cancer-related deaths by 2030 (*1*). Hepatocellular carcinoma (HCC) is the major form of liver cancer and develops in patients with chronic liver conditions, including viral hepatitis, as well as alcoholic and nonalcoholic fatty liver disease (*2*). Generally, chronic liver injuries lead to inflammation, stromal activation, regeneration, fibrosis, and cirrhosis before progression to HCC (*3*).

Autophagy (strictly macroautophagy but hereafter referred to simply as autophagy) is a catabolic membrane-trafficking process that serves to deliver cellular constituents including misfolded proteins and damaged organelles to lysosomes for degradation (*4*). There is now clear evidence that autophagy is important in various diseases including neurodegenerative diseases, chronic liver diseases, and cancer (*5*–*7*). The role of autophagy in cancer, however, is complex and not fully understood, with seemingly opposing roles described in different tumors and at different stages of tumor evolution (*8*–*12*). In the early stages of malignant transformation, autophagy removes damaged mitochondria responsible for the production of reactive oxygen species (ROS) (*13*) and prevents genomic instability (*14*), highlighting its role in preventing tumor initiation. Conversely, in established tumors, autophagy not only can adopt a protumorigenic role, for example, by promoting survival under hypoxic conditions (*15*) and supporting invasion and metastasis (*16*), but also can have a tumor-suppressive role by preventing the proliferative outgrowth of disseminated tumor cells from dormant states at metastatic sites (*17*–*19*).

In the liver, autophagy has primarily been described as tumor suppressive (*11*). Liver-specific deletion of the central autophagy-related protein 5 (ATG5) or ATG7 in mice leads to the formation of liver steatosis, inflammation, ROS production, oval cell formation, fibrosis, hepatomegaly, and the development of HCCs (*11*, *20*). In many cases, loss of autophagy causes accumulation of the autophagy adapter protein p62 (*Sqstm1*), and this can influence antioxidant responses by affecting the axis between Kelch-like ECH-associated protein 1 (KEAP1) and nuclear factor (erythroid-derived 2)-like 2 (NRF2) (*21*). In autophagy-deficient livers, studies have shown that p62 accumulation activates the NRF2 signaling pathway to induce metabolic reprogramming, hepatomegaly, and tumorigenesis (*22*, *23*).

The liver is a plastic organ in which cell fate can change upon injuries to regenerate liver function loss. Hepatocytes and cholangiocytes, epithelial cells that form the liver parenchyma and the bile duct, respectively, can transdifferentiate into one another to reestablish bile duct or liver parenchyma functions (*24*, *25*), with hepatocytes being the primary source of liver regeneration upon injury. Following chronic injury, ductular cells develop in the liver parenchyma when hepatocyte or cholangiocyte function is severely impaired, a process called ductular reaction (*26*). The ductular reaction is a repair mechanism for generating new hepatocytes or cholangiocytes, depending on which liver cells are injured (*27*). However, the origin of the ductular reaction and its role in liver tumorigenesis are controversial with reports indicating that ductular cells can arise from cholangiocyte expansion (*28*, *29*) or through hepatocyte dedifferentiation (*30*, *31*) and reports concluding that the ductular reaction is involved in forming HCC (*32*, *33*), while other studies report the opposite (*34*, *35*). Autophagy-deficient livers undergo a ductular reaction (*36*), and we considered this as an excellent system in which to explore its origin and the role, this phenomenon plays in tumorigenesis.

In this study, we report that autophagy prevents hepatocyte dedifferentiation into ductular liver progenitor cells (LPCs). This ductular LPC population affects HCC formation in autophagy-deficient livers. Mechanistically, we show that autophagy deletion activates both yes-associated protein (YAP) and transcriptional coactivator with PDZ-binding motif (TAZ) in hepatocytes, which are connected to the ductular reaction leading, ultimately, to tumorigenesis. We show that YAP/TAZ coexpression is required to trigger the ductular reaction and tumorigenesis in autophagy-deficient livers.

## RESULTS

### Phosphatase and tensin homolog deficiency accelerates the establishment of a tumor-prone microenvironment in autophagy-deficient livers

**Fig. 1:**
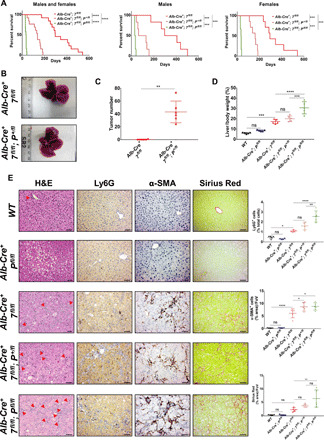
*Pten* deletion accelerates hepatomegaly, the establishment of a tumor-prone microenvironment, and tumorigenesis in Atg7-deficient livers. (**A**) Kaplan-Meier analysis comparing overall survival of mice between males and females (left), males only (middle), or females only (right) (*n* = 6 males and *n* = 7 females per group). Data were analyzed by log-rank Mantel Cox test (***P < 0.001 and ****P < 0.0001). (**B**) Macroscopic pictures from a representative *Alb-Cre^+^; Atg7^fl/fl^* (*Alb-Cre^+^; 7^fl/fl^*) (top) and *Alb-Cre^+^; Atg7^fl/fl^; Pten^+/fl^* (*Alb-Cre^+^; 7^fl/fl^; P^+/fl^*) (bottom) liver in 140-day-old mice. (**C**) Quantification of tumor numbers in *Alb-Cre^+^; 7^fl/fl^* and *Alb-Cre^+^; 7^fl/fl^; P^+/fl^* at 140 days. Data are means ± SD of six mice per group and were analyzed by Mann-Whitney test (**P < 0.01). (**D**) Liver-to-body weight ratio in 4- to 5-week-old mice. Data are means ± SD of five mice per group and were analyzed by one-way analysis of variance (ANOVA) with Tukey correction for multiple comparison tests (***P < 0.001 and ****P < 0.0001). Please note that data are the same controls for *WT* and *Alb-Cre^+^; P^fl/fl^* mice as shown in fig. S1D. (**E**) Hematoxylin and eosin (H&E) staining and immunohistochemical (IHC) analysis of neutrophil recruitment (Ly6G), hepatic stellate cell activation (α-SMA), and collagen deposition (Sirius Red) on paraffin-embedded sections of livers from 4- to 5-week-old mice. Red arrowhead represents ductular structures. Scale bars, 50 μm. Left: Representative staining. Right: Quantifications. Data are means ± SD of four or five mice per group and were analyzed by one-way ANOVA with Tukey correction for multiple comparison tests (*P < 0.05, **P < 0.01, and ****P < 0.0001). All data points are the mean from five pictures per mouse. FoV, field of vision. Please note that data are the same controls for *WT* and *Alb-Cre^+^; P^fl/fl^* mice as shown in fig. S1 (E to G).

Autophagy loss in the murine liver results in hepatomegaly, inflammation, and fibrosis leading to the formation of liver HCCs at 12 months of age (*20*). Phosphatase and tensin homolog (PTEN) expression is lost in approximately half of human liver cancers, and hepatic *Pten*-deficient mice develop HCC at 74 weeks (*37*). To accelerate the autophagy phenotype in the liver, we used the liver-specific promoter *Albumin-Cre* to selectively delete either *Atg7^flox/flox^* or *Atg5^flox/flox^* in the liver in combination with either heterozygous *Pten^+/flox^* (*Alb-Cre^+^; Atg7^fl/fl^; Pten^+/fl^* or *Alb-Cre^+^; Atg5^fl/fl^; Pten^+/fl^)* or homozygous *Pten^flox/flox^* (*Alb-Cre^+^; Atg7^fl/fl^; Pten^fl/fl^* or *Alb-Cre^+^; Atg5^fl/fl^; Pten^fl/fl^*). The reduced gene dosage of *Pten* in an autophagy-deficient background significantly decreased mouse life span similarly in males and females (Fig. 1A and fig. S1A). At end point, while *Alb-Cre^+^; Atg7^fl/fl^; Pten^+/fl^* and *Alb-Cre^+^; Atg5^fl/fl^; Pten^+/fl^* mice developed liver HCCs (Fig. 1B and fig. S1B), *Alb-Cre^+^; Atg7^fl/fl^; Pten^fl/fl^* and *Alb-Cre^+^; Atg5^fl/fl^; Pten^fl/fl^* mice were culled because of extensive hepatomegaly and did not form tumors. To evaluate whether the decreased survival of *Alb-Cre^+^; Atg7^fl/fl^; Pten^+/fl^* and *Alb-Cre^+^; Atg5^fl/fl^; Pten^+/fl^* mice was a result of an early tumor onset, we compared the tumorigenesis of *Pten^+/+^* and *Pten^+/fl^* mice with an autophagy-deficient background at 140 days. This revealed that heterozygous deletion of *Pten* significantly accelerated tumorigenesis in autophagy-deficient livers (Fig. 1, B and C, and fig. S1, B and C). Although conditional double knockout mice did not develop HCC at end point (4 to 5 weeks), they presented with excessive liver overgrowth. When we compared the liver size in 4- to 5-week-old mice, we observed that PTEN loss significantly increased the hepatomegaly of autophagy-deficient livers (Fig. 1D and fig. S1D).

Next, we assessed whether PTEN loss promotes early development of a tumor-permissive microenvironment in 4- to 5-week-old autophagy-deficient livers by looking for markers of inflammation (*38*) and fibrosis. This showed that both hemizygous and homozygous *Pten* deletion significantly increased the recruitment of Ly6G^+^ neutrophils (Fig. 1E and fig. S1E) and activated α–smooth muscle actin^+^ (α-SMA^+^) expressing hepatic stellate cells (Fig. 1E and fig. S1F) in the parenchyma of autophagy-deficient livers, concomitant with a significantly enhanced collagen deposition (Fig. 1E and fig. S1G). PTEN deficiency in 4- to 5-week-old autophagy-competent livers (*Alb-Cre^+^; P^fl/fl^*) did not result in hepatomegaly, inflammation, hepatic stellate cell activation, or fibrosis (Fig. 1, D and E, and fig. S1, D to G). Together, our data suggest that PTEN loss accelerates the early formation of a tumor-prone microenvironment (inflammation, hepatic stellate cell activation, and fibrosis) and tumorigenesis in autophagy-deficient livers.

### Autophagy prevents hepatocyte dedifferentiation into ductular LPCs

**Fig. 2:**
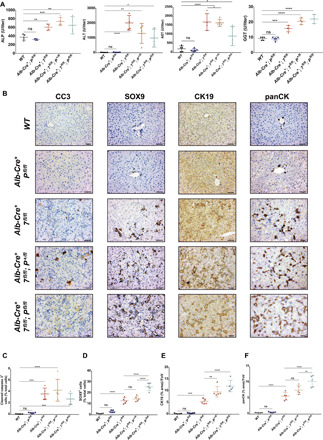
*Pten* deletion accentuates liver damage and the ductular reaction in ATG7-deficient livers. (**A**) Serum analysis of the liver damage markers ALP, ALT, AST, and GGT levels in 4- to 5-week-old mice. Data are means ± SD of three to five mice per group and were analyzed by one-way ANOVA with Dunnett correction for multiple comparison tests (*P < 0.05, **P < 0.01, ***P < 0.001, and ****P < 0.0001). Please note that data are the same controls for *WT* and *Alb-Cre^+^; P^fl/fl^* mice as shown in fig. S2 (A to D). (**B**) IHC analysis of cell death (CC3) and the duct markers SOX9, CK19, and panCK on paraffin-embedded sections of livers from 4- to 5-week-old mice. Scale bars, 50 μm. (**C** to **F**) Quantification of CC3 (C), SOX9 (D), CK19 (E), and panCK (F) from (B). Data are means ± SD of five mice per group and were analyzed by one-way ANOVA with Tukey correction for multiple comparison tests (**P < 0.01, ***P < 0.001, and ****P < 0.0001). All data points are the mean from five pictures per mouse. Please note data are the same controls for *WT* and *Alb-Cre^+^; P^fl/fl^* mice as shown in fig. S2 (E to H).

Following histological examination, we observed an accumulation of atypical ductular structures in the parenchyma of conditional double knockout livers (Fig. 1E), called ductular reaction. Under normal conditions, the liver has ductular structures, called the bile duct, that are formed out of cholangiocytes (Fig. 1E). The ductular reaction is a regeneration program that occurs in the liver following chronic liver injury that impairs the hepatocyte capacity to regenerate the liver (*27*). To evaluate whether hepatocytes are injured upon loss of autophagy, we first assessed the expression of enzymes for liver damage in the serum of 4- to 5-week-old livers. All autophagy-deficient livers had a significant increase in alkaline phosphatase (ALP), alanine aminotransferase (ALT), aspartate aminotransferase (AST) and γ-glutamyl transferase (GGT) levels in comparison to wild-type (WT) (*Alb-Cre^+^; Atg7^+/+^ or Atg5^+/+^; Pten^+/+^*) mice (Fig. 2A and fig. S2, A to D). In addition, we determined whether hepatocytes were dying in our model by looking for cells positive for cleaved caspase 3 (CC3), a marker of apoptosis. We noted a significant augmentation of CC3^+^ hepatocytes in 4- to 5-week-old autophagy-deficient livers when compared to WT livers (Fig. 2, B and C, and fig. S2E), indicating that autophagy prevents hepatocyte cell death. Next, we observed a significant accumulation of the ductular markers sex-determining region Y-box 9 (SOX9), cytokeratin-19 (CK19), and panCK in *Alb-Cre^+^; Atg7^fl/fl^; Pten^fl/fl^* or *Alb-Cre^+^; Atg5^fl/fl^; Pten^fl/fl^* livers in comparison to *Alb-Cre^+^; Atg7^fl/fl^;* or *Alb-Cre^+^; Atg5^fl/fl^* single knockout counterparts (Fig. 2, B and D to F, and fig. S2, F to H), confirming that the ductular reaction is occurring in our accelerated model.

**Fig. 3:**
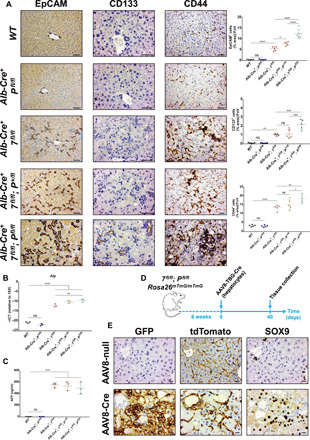
ATG7 prevents hepatocyte dedifferentiation into LPCs. (**A**) IHC analysis of the liver stem cell markers EpCAM, CD133, and CD44 on paraffin-embedded sections of livers from 4- to 5-week old mice. Left: Representative staining. Scale bars, 50 μm. Right: Quantifications. Data are means ± SD of five mice per group and were analyzed by one-way ANOVA with Tukey correction for multiple comparison tests (*P < 0.05, **P < 0.01, ***P < 0.001, and ****P < 0.0001). All data points are the mean were from five pictures per mouse. Please note that data are the same controls for *WT* and *Alb-Cre^+^; P^fl/fl^* mice as shown in fig. S3 (A to C). (**B**) Quantitative reverse transcription polymerase chain reaction (RT-PCR) analysis of *Afp* mRNA isolated from 4- to 5-week-old livers. 18*S* was used as the internal amplification control. Data are means ± SD of three mice per group and were analyzed by one-way ANOVA with Tukey correction for multiple comparison tests (**P < 0.01 and ****P < 0.0001). All data points are the mean from technical triplicates. CT, cycle threshold. (**C**) Enzyme-linked immunosorbent assay (ELISA) analysis of AFP from the serum of 4- to 5-week-old mice. Data are means ± SD of three mice per group and were analyzed by one-way ANOVA with Dunnett correction for multiple comparison tests (****P < 0.0001). All data points are the mean from technical triplicates. (**D**) Schematic representation of the lineage tracing experiment for ductular origin. Eight-week-old *Atg7^flox/flox^; Pten^flox/flox^; Rosa26^mTmG/mTmG^* mice were infected with hepatocyte-specific Cre-expressing adenovirus (AAV8-TBG-Cre) and aged for 40 days. *Rosa26^mTmG^*, *Rosa26^LoxP-Tomato-Stop-LoxP-GFP^*. (**E**) Representative IHC analysis of GFP, tdTomato and SOX9 staining on paraffin-embedded serial sections of liver from *Atg7^flox/flox^; Pten^flox/flox^; Rosa26^mTmG/mTmG^* mice 40 days after infection with AAV8-Cre or the vehicle control (AAV8-null). Scale bars, 20 μm.

As the ductular reaction is a regenerative process for the de novo generation of hepatocytes upon chronic liver injury (*28*–*31*), we hypothesized that ductular cells in our model are LPCs forming to repair injured hepatocytes. To test this, we first looked at the expression of liver stem cell markers in *Atg-* and *Pten-*deficient livers and found increased levels of epithelial cell adhesion molecule (EpCAM), CD133, and CD44 within ductular cells (Fig. 3A and fig. S3, A to C) of autophagy-deficient livers. The expression of the stem cell makers was autophagy dependent but PTEN independent (Fig. 3A and fig. S3, A to C), although *Pten* deletion appears to exacerbate the phenotype caused by *Atg5* or *Atg7* deletion. In addition, we assessed the expression of a-fetoprotein (AFP), a fetal marker reexpressed during HCC and liver stem cell regeneration (*39*). We observed a significant increase in *Afp* mRNA levels (Fig. 3B and fig. S3D) and AFP protein level in the serum (Fig. 3C and fig. S3E) of autophagy-deficient mice when compared to WT counterparts.

We were interested to know how the ductular-reactive cells were forming within the liver parenchyma. It has been established that ductular-reactive cells can originate from dedifferentiated hepatocytes in the parenchyma (*30*, *31*) or from the activation and the proliferation of hepatic progenitor cells from the canal of Hering to regenerate the liver parenchyma when the regenerative function of hepatocytes is impaired (*29*). To determine the cell of origin for the ductular-reactive cells in our model, we crossed *Alb-Cre^−^; Atg7^fl/fl^; Pten^fl/fl^* or *Alb-Cre^−^; Atg5^fl/fl^; Pten^fl/fl^* mice with the double reporter *Rosa26^LoxP-Tomato-LoxP-GFP^* (*Rosa26^mTmG^*) and caused Cre-mediated recombination only in hepatocytes using the *AAV8-TBG-Cre* adeno-associated virus (AAV) (Fig. 3D and fig. S3F), where the Cre recombinase is expressed under the hepatocyte-specific *thyroxine binding globulin (TBG)* promoter (*29*). Following recombination, green fluorescent protein (GFP) will only be expressed in hepatocytes at the membrane, while non-recombined cells and unaffected tissues will remain Tomato^+^. Using this approach, we found that SOX9^+^ ductular-reactive cells expressed GFP at the membrane 40 days following AAV8-Cre infection in autophagy-deficient livers (Fig. 3E), confirming the hepatocyte origin of the ducts (fig. S3G). Together, our data establish that autophagy prevents dedifferentiation of hepatocytes into ductular LPCs.

### HCCs originate from ductular LPCs in autophagy-deficient livers

**Fig. 4:**
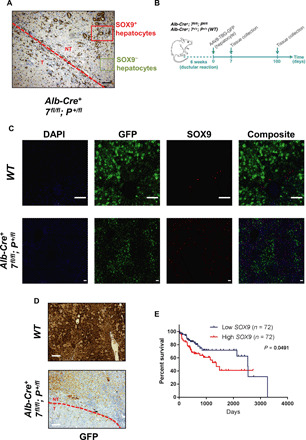
ATG7-deficient tumors originate from hepatocytes-derived LPCs. (**A**) IHC analysis of the duct marker SOX9 on *Alb-Cre^+^; Atg7^fl/fl^; Pten^+/fl^* livers from 140-day-old mice. The red dashed line separates tumor (T) from normal tissue (NT) in the liver. Red and green rectangles outline SOX9^+^ and SOX9^−^ region in normal tissue, respectively. Scale bar, 100 μm. (**B**) Schematic representation of lineage tracing for tumor origin. Six-week-old *Alb-Cre^+^; Atg7^fl/fl^; Pten^+/fl^* and *WT* mice were infected with hepatocyte-specific GFP-expressing adenovirus (AAV8-TBG-GFP) and aged for either 7 or 100 days. (**C**) Immunofluorescence (IF) analysis of GFP and SOX9 on *Alb-Cre^+^; Atg7^fl/fl^; Pten^+/fl^* and *WT* livers 7 days following AAV8-TBG-GFP infection. 4′,6-diamidino-2-phenylindole (DAPI) stains nuclei. Scale bars, 75 μm. (**D**) IHC analysis of GFP on *Alb-Cre^+^; Atg7^fl/fl^; Pten^+/fl^* or *WT* livers 100 days following AAV8-TBG-GFP infection. The red dashed line separates tumor from normal tissue in the liver. Scale bars, 100 μm. (**E**) Kaplan-Meier analysis comparing overall survival between high and low *SOX9* mRNA expression in human liver cancer data (The Cancer Genome Atlas Liver Hepatocellular Carcinoma). Each group represents 20th lower and 20th higher percentile (*n* = 72 per group).

ATG7-deficient livers develop HCCs at around 1 year of age (*20*). Since the ductular reaction is an early event following autophagy inhibition to regenerate the liver and ductular reactive cells express stem cell markers (Fig. 3A and fig. S3, A to C) found in cancer stem cells from HCC (*40*), we hypothesized that ductular LPCs form HCCs in autophagy-deficient livers. To test this, we first assessed whether autophagy-deficient HCCs retain the expression of the duct marker SOX9, and we noted the presence of two distinct hepatocyte populations (SOX9^+^ and SOX9^−^) in the normal region surrounding liver HCCs, with SOX9^+^ hepatocytes found adjacent to ductular structures (Fig. 4A). We found that hepatocytes forming HCCs preserved the ductular marker SOX9 (Fig. 4A). To further evaluate the role of the ductular reaction in tumorigenesis, we infected *Alb-Cre^+^; Atg7^fl/fl^; Pten^+/fl^* and *WT* mice with the AAV8-TBG-GFP adenovirus at 6 weeks of age to label hepatocytes with GFP (Fig. 4B). At this age, the ductular reaction is occurring in autophagy-deficient livers, which allows us to distinguish and discriminate between resident hepatocytes (GFP^+^) and ductular reactive cells (GFP^−^) following AAV8-TBG-GFP infection to trace their role in tumorigenesis. First, we confirmed that at 7 days after AAV8-TBG-GFP infection, SOX9^+^ LPCs were GFP^−^, while hepatocytes (SOX9^−^) expressed GFP in autophagy-deficient livers (Fig. 4C), confirming that ductular LPCs are not expressing GFP following AAV8-TBG-GFP infection. We then assessed the expression of GFP in autophagy-deficient HCCs 100 days after AAV8-TBG-GFP infection. This revealed that tumors forming in *Alb-Cre^+^; Atg7^fl/fl^; Pten^+/fl^* livers expressed no GFP in comparison to the surrounding normal hepatocytes, which retained GFP expression (Fig. 4D), highlighting that the ductular cells initiate tumorigenesis in autophagy-deficient livers. We also found that high expression of *SOX9* correlates with a decreased survival in human HCCs (Fig. 4E). Together, our data establish that ductular LPCs, formed early upon autophagy deficiency, ultimately lead to the generation of HCCs in autophagy-deficient livers.

### Autophagy loss in the liver enhances a YAP/TAZ signature within ductular LPCs

**Fig. 5:**
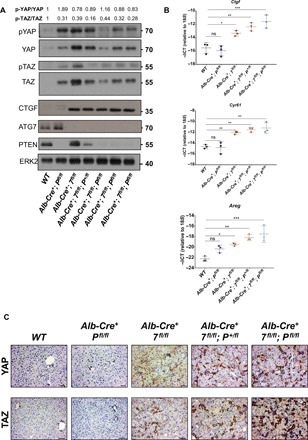
YAP and TAZ are active in LPCs of ATG7-deficient livers. (**A**) Immunoblotting analysis of phosphorylated YAP (p-YAP), total YAP, phosphorylated TAZ (p-TAZ), total TAZ, CTGF, ATG7, and PTEN from 4- to 5-week-old total liver lysates. Extracellular signal–regulated kinase 2 (ERK2) was used as the loading control. (**B**) Quantitative RT-PCR analysis of the YAP/TAZ targets *Ctgf*, *Cyr61*, and *Areg* mRNA isolated from 4- to 5-week-old livers. 18*S* was used as the internal amplification control. Data are means ± SD of three mice per group and were analyzed by one-way ANOVA with Dunnett correction for multiple comparison tests (*P < 0.05, **P < 0.01, and ***P < 0.001). All data points are the mean from technical triplicates. (**C**) IHC analysis of YAP and TAZ on paraffin-embedded sections of livers from 4- to 5-week-old mice. Scale bars, 50 μm.

Blocking the formation of the ductular reaction would be beneficial in preventing human HCC (*41*). YAP and TAZ are transcriptional coactivators essential in controlling organ size (*42*), hepatocyte dedifferentiation (*31*), stemness (*43*), and liver tumorigenesis (*44*, *45*). The Hippo pathway regulates the activation of YAP and TAZ, and phosphorylation of both coactivators primes them for degradation. As our autophagy-deficient liver model develops severe hepatomegaly (Fig. 1D and fig. S1D), dedifferentiates hepatocytes into ductular LPCs (Figs. 2 and 3 and figs. S2 and S3), and induces tumorigenesis, we next investigated whether YAP and TAZ are active in early-stage autophagy-deficient livers exhibiting ductular reaction. First, we compared the protein levels of the inactive forms of YAP and TAZ (phosphorylated YAP and phosphorylated TAZ), with the levels of total YAP and total TAZ (active forms) in 4- to 5-week-old livers (Fig. 5A). We noticed that the ratio of phosphorylated YAP and phosphorylated TAZ was reduced in autophagy-deficient livers in comparison to WT counterparts (Fig. 5A), highlighting that unphosphorylated YAP and unphosphorylated TAZ accumulate in autophagy-deficient livers undergoing ductular reaction.

To evaluate whether YAP and TAZ are functionally active in autophagy-deficient livers, we tested for the expression of YAP/TAZ transcriptional targets in 4- to 5-week-old livers. We found that mRNA levels of connective tissue growth factor (*Ctgf*), amphiregulin (*Areg*), and cysteine-rich angiogenic inducer 61 (*Cyr61*), three YAP/TAZ target genes (*46*, *47*), were all significantly up-regulated in autophagy-deficient livers (Fig. 5B and fig. S4A). At the protein level, CTGF was increased in total liver lysates of all autophagy-deficient conditions (Fig. 5A). Next, we assessed the localization of YAP and TAZ in 4- to 5-week-old autophagy-deficient livers and observed that both YAP and TAZ strongly accumulated in the ductular cells, whereas YAP and TAZ were found in the bile duct and the canal of Hering of WT counterparts (Fig. 5C and fig. S4B). Collectively, our data therefore indicate that autophagy loss in hepatocytes triggers a YAP/TAZ signature within the ductular LPC population.

### TAZ is not degraded by autophagy

YAP is turned over not only by the proteasome (*48*, *49*), but also by autophagy as shown in recent reports (*20*, *50*). As TAZ is a YAP homolog, we next wondered whether TAZ accumulation and activation in our autophagy-deficient livers were due to blockage of autophagy-mediated degradation of TAZ. To test more directly whether TAZ is degraded by autophagy, we first deleted ATG7 or ATG5 expression in the liver cancer cell lines HLE and Huh7 using the CRISPR-Cas9–mediated gene disruption system. Next, we treated each cell line with Earle’s balanced salt solution (EBSS), to induce starvation-mediated autophagy, in combination with or without 200 nM bafilomycin A1 (Baf) for 2 hours to prevent lysosomal degradation of autophagosomes. We checked for the efficient disruption of ATG7 or ATG5 expression following lenti-CRISPR infection in HLE (fig. S5A) and Huh7 (fig. S5B), and we analyzed the conversion of microtubule-associated protein 1A/1B-light chain 3 (LC3)–I (diffuse form in the cytosol) into LC3-II (lipidated form attached to autophagosomes), to confirm loss of autophagy. Examination of TAZ revealed that its levels did not change upon starvation-induced autophagy (EBSS), blockage of lysosomal autophagy degradation [Dulbecco’s modified Eagle’s medium (DMEM) + Baf and EBSS + Baf], or disruption of *ATG7*/*ATG5* (ATG7^CRISPR^/ATG5^CRISPR^) in HLE and Huh7 cells (fig. S5, A and B). Unexpectedly, we also observed that not only YAP levels accumulated under EBSS only and EBSS and Baf conditions but also this occurred in ATG7^CRISPR^/ATG5^CRISPR^ cells, indicating that this was an autophagy-independent effect. Together, our data indicate that TAZ and YAP are not directly turned over by autophagy in liver cells and that the accumulation of YAP and TAZ in autophagy-deficient livers is not the result of the inhibition of the autophagy degradation pathway but instead is due to the expansion of ductular cells in vivo, which are known to express YAP and TAZ (Fig. 5 and fig. S4) (*51*).

### YAP and TAZ deletion blocks ductular cell formation and tumorigenesis in autophagy-deficient livers

**Fig. 6:**
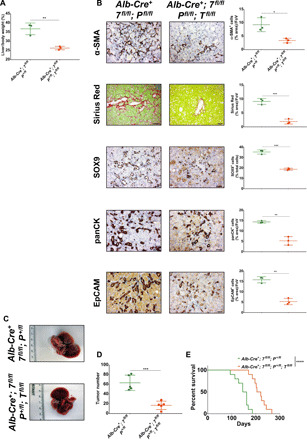
TAZ deletion impairs hepatomegaly, hepatic stellate cell activation, fibrosis, and tumorigenesis in ATG7-deficient livers. (**A**) Liver-to-body weight ratio in 4- to 5-week-old mice. Data are means ± SD of three mice per group and were analyzed by unpaired two tailed *t* test (**P < 0.01). (**B**) IHC analysis of hepatic stellate cell activation (α-SMA), collagen deposition (Sirius Red), duct markers (SOX9 and panCK), and liver stem cell marker EpCAM on paraffin-embedded sections of livers from 4- to 5-week-old mice. Scale bars, 50 μm. Left: Representative staining. Right: Quantifications. Data are mean ± SD of three mice per group and were analyzed by unpaired two-tailed *t* test (*P < 0.05, **P < 0.01, and ***P < 0.001). All data points are the mean from five pictures per mouse. (**C**) Macroscopic pictures of *Alb-Cre^+^; Atg7^fl/fl^; Pten^+/fl^* (top) and *Alb-Cre^+^; Atg7^fl/fl^; Pten^+/fl^; Taz^fl/fl^* (*Alb-Cre^+^; 7^fl/fl^; P^+/fl^; T^−/−^*) (bottom) liver in 140-day-old mice. (**D**) Quantification of tumor numbers in *Alb-Cre^+^; Atg7^fl/fl^; Pten^+/fl^* and *Alb-Cre^+^; Atg7^fl/fl^; Pten^+/fl^; Taz^fl/fl^* at 140 days. Data are means ± SD of five mice per group and were analyzed by unpaired two-tailed *t* test (***P < 0.001). (**E**) Kaplan-Meier analysis comparing overall survival between *Alb-Cre^+^; Atg7^fl/fl^; Pten^+/fl^* and *Alb-Cre^+^; Atg7^fl/fl^; Pten^+/fl^; Taz^fl/fl^* mice (*n* = 5 males and *n* = 5 females per group). Data were analyzed by log-rank Mantel-Cox test (****P < 0.0001).

Deletion of YAP partially rescued hepatomegaly, fibrosis, and tumorigenesis induced by autophagy blockage in the liver (*20*). As a YAP homolog, TAZ can compensate YAP activity if the latter is lost (*52*). Since we observed in our model that YAP and TAZ are activated within the ductular LPC population, we hypothesized that deleting both YAP and TAZ might prevent the early ductular reaction and subsequent HCC formation in autophagy-deficient livers. First, we evaluated whether TAZ has a role in the phenotype of autophagy-deficient livers. To test this, we crossed *Wwtr1^flox/flox^* (encoding TAZ) mice (*53*) with our liver-specific autophagy-deficient model, and we observed that loss of TAZ significantly reduced liver size of 4- to 5-week-old autophagy-deficient livers (Fig. 6A and fig. S6A). Next, we found that TAZ loss also significantly reduced the accumulation of activated α-SMA^+^ hepatic stellate cells and collagen deposition in 4- to 5-week-old autophagy-deficient livers (Fig. 6B and fig. S6B), indicating that TAZ contributes to hepatic stellate cell activation and fibrosis in our model. In addition, TAZ loss significantly decreased SOX9^+^, panCK^+^, and EpCAM^+^ cells in 4- to 5-week-old autophagy-deficient livers (Fig. 6B and fig. S6B), highlighting that TAZ loss hinders the formation of ductular LPCs upon autophagy deficiency in the liver. We next compared tumor formation between *Alb-Cre^+^; Atg7^fl/fl^; Pten^+/fl^* or *Alb-Cre^+^; Atg5^fl/fl^; Pten^+/fl^* and *Alb-Cre^+^; Atg7^fl/fl^; Pten^+/fl^; Taz^fl/fl^* or *Alb-Cre^+^; Atg5^fl/fl^; Pten^+/fl^; Taz^fl/fl^* in 140-day-old livers and noted that TAZ deletion caused a highly significant decrease in tumorigenesis in autophagy-deficient livers (Fig. 6, C and D, and fig. S6, C and D) that was accompanied by a significant increase in the survival of autophagy-deficient mice (Fig. 6E and fig. S6E). Last, we evaluated whether TAZ has a role in the proliferation of ductular LPCs. We found that TAZ loss did not impair the number of Ki-67^+^ proliferative LPCs in 4- to 5-week-old autophagy-deficient livers (fig. S7).

**Fig. 7:**
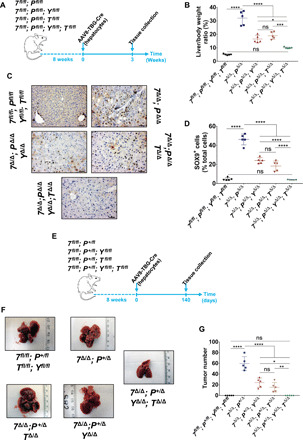
YAP and TAZ cooperate to drive the duct-induced tumorigenesis in ATG7-deficient livers. (**A**) Schematic representation. Eight-week old *Atg7^fl/fl^; Pten^fl/fl^* ± *Yap^fl/fl^* (*Y^fl/fl^*) and/or *Taz^fl/fl^* (*T^fl/fl^*) mice were infected with AAV8-TBG-Cre and aged for 3 weeks before hepatomegaly and ductular reaction analysis. (**B**) Liver-to-body weight ratio in mice 3 weeks after AAV8 infection. Data are means ± SD of five mice per group and were analyzed by one-way ANOVA with Tukey correction for multiple comparison tests (*P < 0.05, ***P < 0.001, and ****P < 0.0001). (**C**) IHC analysis of the duct marker SOX9 on paraffin-embedded sections of livers from mice 3 weeks after AAV8 infection. Scale bars, 50 μm. (**D**) Quantification of SOX9 from (C). Data are means ± SD of five mice per group and were analyzed by one-way ANOVA with Tukey correction for multiple comparison tests (****P < 0.0001). All data points are the mean from five pictures per mouse. (**E**) Schematic representation. Eight-week-old *Atg7^fl/fl^; Pten^+/fl^; Yap^fl/fl^* and/or *Taz^fl/fl^* mice were infected with AAV8-TBG-Cre and aged for 140 days before tumor analysis. (**F**) Macroscopic pictures from 140 days after AAV8-Cre livers. (**G**) Quantification of tumor numbers in 140 days after AAV8-Cre livers. Data are means ± SD of five mice per group and were analyzed by one-way ANOVA with Tukey correction for multiple comparison tests (*P < 0.05, **P < 0.01, and ****P < 0.0001). All data points are the mean from five pictures per mouse. *X^fl/fl^*, AAV8-null infected; *X*^Δ*/*Δ^, AAV8-Cre infected.

To evaluate whether there was any redundancy between YAP and TAZ in our model, we crossed *Yap1^flox/flox^* mice (*53*) to our liver-specific (*Alb-Cre*) autophagy- and TAZ-deficient model to evaluate the effect of YAP/TAZ double knockout on the ductular reaction and tumorigenesis of autophagy-deficient livers. Unexpectedly, we observed that 40% (9 of 22 mice) of YAP-deficient mice developed jaundice within 6 to 8 weeks regardless of *Atg7*, *Atg5*, *Pten*, or *Wwtr1* genotype. This is likely because YAP is highly expressed in the bile duct of WT mice (Fig. 5C and fig. S4B), and the *Albumin* promoter driving Cre recombinase expression is expressed in hepatoblasts, the embryonic progenitor cells generating hepatocytes and cholangiocytes (*54*). YAP deletion in our *Albumin-Cre* model can therefore impair cholangiocyte function in the bile duct leading to acute jaundice. To overcome this phenotype for long term studies, we used AAV8-TBG-Cre adenovirus to induce Cre recombination more specifically in the hepatocytes of our *Atg7^flox/flox^; Pten^flox/flox^; Yap ^flox/flox^; Taz^flox/flox^* model (Fig. 7A). First, we assessed the effect of YAP/TAZ deletion on the hepatomegaly and ductular reaction of autophagy-deficient livers 3 weeks following AAV8-TBG-Cre recombination and confirmed the recombination of *Atg7*, *Pten*, *Yap*, and *Wwtr1* alleles in AAV8-TBG-Cre–infected livers (fig. S8). We found that although YAP or TAZ deletion significantly reduced hepatomegaly of autophagy-deficient livers (Fig. 7B), YAP/TAZ double knockout mice significantly restored liver size to that observed in nonrecombined counterparts infected with the *AAV8-TBG-null* adenovirus (Fig. 7B). In addition, we noted that while the individual deletion of *Yap* or *Taz* significantly impaired the formation of SOX9^+^ cells in autophagy-deficient livers (Fig. 7, C and D), only YAP/TAZ codeletion completely blocked the formation of SOX9^+^ cells in autophagy-deficient livers (Fig. 7, C and D). In this *AAV8-TBG-Cre* model, *Atg7*^Δ*/*Δ^*; Pten*^Δ*/*Δ^ mice had to be culled because of hepatomegaly and did not develop tumors at humane end point. To evaluate the role of YAP/TAZ loss in the tumorigenesis of autophagy-deficient livers, we infected *Atg7^flox/flox^; Pten^+/flox^; Yap^flox/flox^; Taz^flox/flox^* with AAV8-TBG-Cre adenovirus and assessed tumor formation 140 days following AAV8 infection (Fig. 7E). We observed that while *Yap* or *Taz* deletion significantly impaired tumorigenesis in autophagy-deficient livers (Fig. 7, F and G), only YAP/TAZ codeletion completely prevented tumor formation (Fig. 7, F and G). Our data therefore show that deleting YAP and TAZ suppresses the ductular reaction and tumorigenesis of autophagy-deficient livers. However, in this context, we observed functional redundancy between YAP and TAZ, and only the combined deletion of both these genes could revert the effects on tissue overgrowth and tumor development.

## DISCUSSION

We report a new model for extensive ductular reaction upon deletion of ATG5 or ATG7 and PTEN in the murine liver. Although *Pten*-deficient livers develop steatosis and HCC (*37*), we observed that hepatic Pten deletion alone did not initiate liver damage, inflammation, hepatic stellate cell activation, fibrosis, or a ductular reaction in young livers, but these effects were observed on hepatic deletion of ATG5 or ATG7. ATG5 and ATG7 are two proteins that are essential for the stage of autophagy that involves LC3 conjugation. ATG5 and ATG7 are also important for two other processes that involve the LC3 conjugation machinery: LC3-associated phagocytosis (LAP) (*55*) and LC3-associated endocytosis (LANDO) (*56*). We consider, however, that the core observations in our study relating to tumor development and liver injury are connected to autophagy, as previous studies have shown that they can be reversed by concomitant deletion of the autophagy adapter protein p62 (*11*, *22*, *57*), and autophagy adapter proteins are not thought to be involved in LAP or LANDO (*58*). We cannot fully discount that some of the effects we observe on deletion of ATG5 or ATG7 may be related to LAP or LANDO rather than autophagy or a combination thereof. Future studies to clarify this point using deletion of other factors such as FIP200 or ATG13 that are involved in autophagy, but not LAP and LANDO (*59*–*62*), would certainly be merited to investigate this possibility.

Autophagy is impaired in *Pten*-deficient mice due to mTORC1 activation; however, autophagy is not blocked in *Pten*-deficient livers (*63*). LC3 is still conjugated to phosphatidylethanolamine leading to autophagosome and autolysosome formation when Pten expression is lost (*63*). This dictates an important role for autophagy in hepatocytes to prevent the microenvironmental remodeling and ductular reaction in healthy livers, with Pten cooperating with the autophagy-specific phenotype. Pten loss induces cellular senescence to protect from tumorigenesis in different models (*64*, *65*). However, we noticed the presence of apoptotic hepatocytes following autophagy abrogation and Pten deletion. The extent of injury in hepatocytes determines their fate toward senescence or cancer (*66*). Acute injury in hepatocytes results in senescence (*67*), while chronic injury does not activate senescence in hepatocytes, ultimately leading to HCC (*66*). Autophagy degrades damaged mitochondria, a process named mitophagy, to maintain cellular homeostasis. In hepatocytes, loss of autophagy leads to ROS accumulation, damaged mitochondria, and dysfunction (*11*, *22*, *68*, *69*). We suggest that the persistence of chronic damage and defects in damaged mitochondria clearance by mitophagy drive apoptosis and tumorigenesis in our autophagy- and Pten-deficient livers.

In our autophagy- and Pten-deficient model, we observed that following liver injury, hepatocytes dedifferentiate into ductular LPCs. This ductular reactive phenotype is not unique to the loss of autophagy as it has previously been observed in animal models subjected to diet modification, e.g., a diet enriched in 3,5-diethoxycarboncyl-1,4-dihydrocollidine (*70*) or choline-deficient, ethionine-supplemented diet (*71*). This indicates that the ductular reaction is likely to be a secondary effect of autophagy inhibition due to liver damage caused by autophagy loss. The origin of the ductular reaction in rodents is still controversial, with reports indicating the role of biliary cells (*28*, *29*) or hepatocytes (*30*, *31*) in forming LPCs with the capacity for generating new hepatocytes upon liver injury. Here, we show in a genetically modified mouse model that ductular reactive cells arise from mature hepatocytes upon injury induced by autophagy deficiency. The cellular plasticity of human hepatocytes can also generate ductular cells in a transplantation mouse model (*30*), strengthening the hepatocyte origin of the ductular reaction in human liver diseases.

The plastic differentiation program of the ductular reaction for liver regeneration is defined by the origin of the injuries. Following bile duct injury, resident LPCs/biliary cells (*26*) and hepatocyte-derived LPCs (*72*) regenerate biliary cells. When hepatocyte function is impaired, resident LPCs/biliary cells (*28*, *29*, *73*) or hepatocyte-derived LPCs (*30*, *31*, *74*) generate new hepatocytes. The decision to recruit biliary cells or hepatocytes during the ductular reaction remains elusive, and future studies will be required to shed further light on this mechanism.

Autophagy loss has been previously shown to give rise to HCC in mice (*20*). Our results suggest that the hepatocyte-derived ductular reaction gives rise to HCC in autophagy-deficient livers. While some studies conclude that the ductular reaction is not involved in liver carcinogenesis (*34*, *35*, *74*), other studies do report a role for the ductular reaction in initiating HCCs (*32*, *33*). Although all these studies recombine LPCs for lineage tracing, they differ with respect to the timing between the induction of LPC labeling and the start of the injury. Recombination of LPCs for lineage tracing before inducing liver injury (*34*, *35*, *74*) does not label hepatocyte-derived LPCs, excluding them from the lineage tracing of HCCs. In contrast, recombination of LPCs for lineage tracing following liver injury results in LPC-derived HCCs (*32*, *33*). In our autophagy- and Pten-deficient model, we report that hepatocyte-derived LPCs generate SOX9^+^ hepatocytes that give rise to HCC. The ability of LPCs to induce tumorigenesis has been controversial since it is generally accepted that HCC originates from hepatocytes. Here, we reconcile these findings by showing that HCC does originate from hepatocytes, but these hepatocytes, early upon liver injury, dedifferentiate into LPCs to attempt to regenerate liver function, before transforming into HCC.

In human liver diseases, the accumulation of LPCs is observed in nonalcoholic steatohepatitis–induced cirrhosis preceding HCC (*75*), and the presence of peritumoral ductular reaction is a poor prognostic factor for human HCC after resection (*76*), indicating the importance of targeting the ductular reaction in human liver diseases. The gene signature of autophagy-deficient mice is similar to the human transcriptomes of nonalcoholic fatty livers (*20*), and rat livers from rats fed a high-fat diet reduce their autophagy function (*77*). Restoring autophagy could therefore be a beneficial treatment in injured livers harboring a ductular reaction.

Mechanistically, we report that YAP and TAZ cooperate to drive hepatocyte dedifferentiation and tumorigenesis in autophagy-deficient livers. Unlike a previous study on YAP (*20*), we uncovered that TAZ also plays a role in promoting hepatomegaly, ductular reaction, stromal activation, fibrosis, and tumorigenesis in autophagy-deficient livers. TAZ deletion alone, similar to YAP deletion alone (*20*), only impaired carcinogenesis in autophagy-deficient livers. However, TAZ loss did not impair the proliferative outgrowth of the ductular LPC population. Here, we speculate that TAZ is involved in the differentiation switch in our model as its homolog YAP can directly drive hepatocyte dedifferentiation (*31*), and, more recently, YAP/TAZ have been described as regulators of stemness and cell plasticity in glioblastoma (*78*). We found that YAP and TAZ are not directly turned over by autophagy and that their accumulation in the absence of autophagy in vivo is associated with the increased presence of ductular cells, which are known to express YAP and TAZ (*79*). YAP and TAZ are mechanosensors and mechanotransducers (*80*), and their activation is linked to the stiffness of the extracellular matrix (*81*). As we noted a significant increase in extracellular matrix remodeling and fibrosis (Fig. 1E and fig. S1, F and G) correlating with a significant increase in YAP^+^/TAZ^+^ ductular LPCs in our models, we suggest that YAP and TAZ are also activated in response to the microenvironment changes following autophagy and PTEN deletion in the liver. Building on these findings, we observed that only the combined deletion of YAP and TAZ prevented the emergence of hepatocyte-derived LPCs that initiate tumorigenesis in autophagy-deficient livers. Our study uncovered a role for autophagy in suppressing the emergence of hepatocyte-derived ductular LPCs that can give rise to HCCs via concomitant activation of YAP and TAZ.

## MATERIALS AND METHODS

### Animal experiments

Male and female animals were housed in a pathogen-free environment and kept under standard conditions with a 12-hour day/night cycle and access to food and water ad libitum. All in vivo experiments were carried out under guidelines approved by the Glasgow University Animal Welfare and Ethical Review Body and in accordance with U.K. Home Office guidelines under license P54E3DD25. As described previously (*82*), *Alb-Cre^+^* mice [RRID (research resource identifier): MGI:2176228] were crossed to *Atg7^fl/fl^* (*68*) (RRID: MGI:3590136) or *Atg5^fl/fl^* (*83*) (RRID: MGI:3612279) and *Pten^fl/fl^* (*84*) (RRID: MGI:2182005) to generate the different combinations on a mixed background. Subsequently, *Atg7^fl/fl^; Pten^fl/fl^* and *Atg5^fl/fl^; Pten^fl/fl^* mice were crossed to *Yap1^fl/fl^; Wwtr1^fl/fl^* (the Jackson laboratory, stock 030532, RRID: IMSR_JAX:030532) (*53*) animals to generate all the different combinations. Experimental cohort (males and females) sizes were based on previous similar studies that have given statistically significant results while also respecting the limited use of animals in line with the 3R system: replacement, reduction, and refinement. All treatment studies were randomized but did not involve blinding. Genotyping was performed by Transnetyx. To lineage trace the ductular cell origin, we crossed our model with the *Rosa26*-mtdTomato-mEGFP mouse (the Jackson laboratory, stock 007576, RRID: IMSR_JAX:007576) (*85*).

In AAV8 studies, AAV8 recombination was performed as previously described (*67*). Briefly, viral particles [2 × 10^11^ genomic copies per mouse] of AAV8.TBG.PI.Cre.rBG (Addgene, catalog no. 107787-AAV8), AAV8.TBG.PI.eGFP.WPRE.bGH (Addgene, catalog no. 105535-AAV8), or AAV8.TBG.PI.Null.bGH (Addgene, catalog no. 105536-AAV8) were injected in 6-week-old (AAV8-GFP and AAV8-null) or 8-week-old (AAV8-Cre and AVV8-null) mice via tail vein in 100 μL of phosphate-buffered saline (PBS).

### Animal tissue harvesting and serum analysis

Mice were euthanized by CO_2_ inhalation followed by cervical dislocation, and blood was harvested by cardiac puncture in accordance with U.K. Home Office guidelines. Tissues were weighed and stored immediately at −80°C or in paraffin blocks after fixation in 10% formalin (in PBS) for 24 hours, followed by dehydration in 70% ethanol before embedding. Blood samples (EDTA-plasma and serum) were stored at −80°C following 10-min centrifugation at 900*g* at 4°C. Serum was sent to the Veterinary Diagnostic Services (University of Glasgow) for ALT, AST, ALP, and GGT analyses.

### Plasma AFP levels

Plasma AFP levels were assessed using the enzyme-linked immunosorbent assay (ELISA) kit (catalog no. ab210969) according to the manufacturer’s instruction. Each sample was analyzed in triplicate.

### Immunohistochemistry and immunofluorescence

For immunohistochemical (IHC) or immunofluorescence (IF) studies, paraffin-embedded sections were deparaffinized, rehydrated, and heated to 95° to 97°C either in Lab Vision Citrate Buffer for heat-induced epitope retrieval (pH 6.0) (Thermo Fisher Scientific, catalog no. 12638286), EnVision FLEX Target Retrieval Solution, High pH (Agilent, catalog no. K8004), BOND Epitope Retrieval Solution 2 (ER2) (Leica, catalog no. AR9640), or Antigen Unmasking Solution, Citric Acid Based (Vector Laboratories, catalog no. H-3300) for antigen retrieval, depending on the primary antibody used. Primary antibodies used for IHC analyses: Ly6G (Bio X Cell, catalog no. BE0075-1, RRID: AB_1107721, rat, ER2; 1:60,000), α-SMA (Sigma-Aldrich, catalog no. A2547, RRID: AB_476701, mouse, citric acid; 1:25,000), CC3 (Asp175, Cell Signaling Technology, catalog no. 9661, RRID: AB_2341188, rabbit, ER2; 1:500), SOX9 (Millipore, catalog no. AB5535, RRID: AB_2239761, rabbit, high pH; 1:500), CK19 (Novus, catalog no. NB100-687, RRID: AB_2265512, rabbit, high pH; 1:100), panCK (Lab Vision, catalog no. MS-343-P, RRID: AB_61531, mouse, Citric acid; 1:100), EpCAM (Abcam, catalog no. ab71916, RRID: AB_1603782, rabbit, high pH; 1:1500), CD133 (Abcam, catalog no. ab19898, RRID: AB_470302, rabbit, citrate pH 6; 1:200), CD44 (BD Biosciences, catalog no. 550538, RRID: AB_393732, rat, ER2; 1:300), GFP (Cell Signaling Technology, catalog no. 2555, RRID: AB_10692764, rabbit, ER2; 1:600), red fluorescent protein (Rockland, catalog no. 600-401-379, RRID: AB_2209751, rabbit, high pH; 1:1000), YAP (Cell Signaling Technology, catalog no. 4912, RRID: AB_2218911, rabbit, high pH; 1:50), WW domain containing transcription regulator 1 (WWTR1)/TAZ (Sigma-Aldrich, catalog no. HPA007415, RRID: AB_1080602, rabbit, high pH; 1:100), and Ki-67 (Cell Signaling Technology, catalog no. 12202, RRID: AB_2620142, rabbit, ER2; 1:1000). Primary antibodies were incubated with sections for 40 min at room temperature or overnight at 4°C. For IHC analysis, primary antibodies were detected using mouse or rabbit EnVision^+^ System kits (Agilent, catalog no. K4001 and K4006) or ImmPRESS horseradish peroxidase (HRP) goat anti-rat immunoglobulin G (IgG) polymer detection kit (Vector Laboratories, catalog no. MP-7404) and 3,3′-diaminobenzidine substrate (Agilent, catalog no. K4011). Slides were then counterstained with hematoxylin solution. Images were obtained on a Zeiss AX10 (light microscopy) at a ×20 or ×40 magnification.

For IF analysis, SOX9/GFP immunofluorescent primary antibodies were applied sequentially. First, slides were incubated with a chicken polyclonal GFP antibody (Abcam, catalog no. ab13970, RRID: AB_300798, citrate; 1:200) overnight at 4°C and was detected using a biotinylated goat anti-chicken (Vector Laboratories, catalog no. BA-9010, RRID: AB_2336114; 1:200) coupled to Avidin-HRP (Vector Laboratories, PK-7100) and a PerkinElmer TSA Plus Cyanine 3 signaling amplification kit (NEL744B001KT; 1:50). This was followed by a second antigen retrieval to denature any antibodies in the tissue. Slides were then incubated with a rabbit monoclonal SOX9 antibody (Abcam, catalog no. ab185230, RRID: AB_2715497, citrate; 1:500) overnight at 4°C and detected using a donkey anti-rabbit Alexa Fluor 488 secondary antibody (Molecular Probes, catalog no. A-21206, RRID: AB_2535792; 1:200). Slides were then counterstained with 4′,6-diamidino-2-phenylindole (DAPI). Images were obtained on a Zeiss 710 confocal microscope at a ×20 magnification. For collagen staining, sections were rehydrated and then immersed in Picro Sirius Red solution [0.1% Direct Red 80 (Sigma-Aldrich, 41496LH) and 0.1% Fast Green FCF (Raymond Lamb, S142-2) diluted in aqueous picric acid solution] for 2 hours.

### Cell culture

HLE and Huh7 were grown in DMEM (Gibco, 21969-035) supplemented by 10% fetal bovine serum (FBS; Gibco, 10270-106), 2 mM glutamine (Gibco, 25030-032), streptomycin (100 μg/ml), and penicillin (100 U/ml; Gibco, 15140-122) (complete DMEM) at 37°C and 5% CO_2_. For starvation-induced autophagy experiments, cells were washed twice in PBS and starved in EBSS (Sigma-Aldrich, E2888) containing or not 200 nM Baf (LC Labs, B-1080) for 2 hours. HLE and Huh7 cell lines were provided by T. Bird.

### Lentivirus production and infection for CRISPR

Lentiviruses were produced using human embryonic kidney (HEK) 293T cells using calcium/phosphate transfection protocol. Cells were transfected overnight with lentiviral, packaging, and envelope plasmids (pPAX2 and pVSVG). The following day, media were replaced by complete DMEM containing 20% FBS for 24 hours. Then, virus-enriched media were collected, filtered (0.45 μm), supplemented with polyprene (4 μg/ml; Sigma-Aldrich, H9268), and transferred to recipient cells. In the meantime, HEK293T cells were kept in DMEM containing 20% FBS for an additional 24 hours to perform a second round of infection of recipient cells as described before. Last, infected cells were selected with puromycin (2 μg/ml; Sigma-Aldrich, P9620) for 10 days. The following single-guide RNA sequences were used in this study: human ATG7, 5′-GAA GCT GAA CGA GTA TCG GC-3′ (*86*); human ATG5, 5′-AAG AGT AAG TTA TTT GAC GT-3′ (*86*); nontargeting control, 5′-GTA GCG AAC GTG TCC GGC GT-3′ (*87*).

### Protein extraction and Immunoblotting

Livers were dissociated using a Precellys Evolution (Bertin Technologies) and lysed in 1% Triton X-100, 0.1% SDS, 50 mM Hepes (pH 7.5), 150 mM NaCl, 100 mM NaF, and 10 mM EDTA, supplemented with Halt protease and phosphatase inhibitor cocktail (Thermo Fisher Scientific, catalog no. 87786). After 15-min centrifugation at 12,000*g* at 4°C, the supernatant was removed, and the concentration of solubilized proteins was determined with the Pierce bicinchoninic acid assay (Thermo Fisher Scientific, catalog no. 23225). Protein lysates were separated by SDS–polyacrylamide gel electrophoresis with Criterion TGX Stain-Free precast gels (Bio-Rad) or the NuPAGE 4 to 12% bis-tris gel (Invitrogen) and blotted onto polyvinylidene difluoride membranes (Merck). Criterion TGX Stain-Free precast gels (Bio-Rad) were activated using the ChemiDoc (Bio-Rad) to detect total protein levels. Total protein level was measured before and after transfer. Western blot analysis was performed according to the manufacturer’s instructions for Criterion TGX Stain-Free precast gels or for the NuPAGE 4 to 12% bis-tris gel (Invitrogen). The following antibodies were used at a dilution of 1:1000 unless otherwise stated: p-YAP (Cell Signaling Technology, catalog no. 13008, RRID: AB_2650553), YAP (Cell Signaling Technology, catalog no. 4912, RRID: AB_2218911; 1:750), p-TAZ (Cell Signaling Technology, catalog no. 59971, RRID: AB_2799578), YAP/TAZ (Cell Signaling Technology, catalog no. 8418, RRID: AB_10950494), CTGF (Abcam, catalog no. ab125943, RRID: AB_2858254), ATG7 (Cell Signaling Technology, catalog no. 8558, RRID: AB_10831194), PTEN (Cell Signaling Technology, catalog no. 9559, RRID: AB_390810), extracellular signal–regulated kinase 2 (ERK2; Santa Cruz Biotechnology, catalog no. sc-154, RRID: AB_2141292), LC3B (Cell Signaling Technology, catalog no. 2775, RRID: AB_915950), ATG5 (Cell Signaling Technology, catalog no. 12994, RRID: AB_2630393), glyceraldehyde-3-phosphate dehydrogenase (Abcam, catalog no. ab9485, RRID: AB_307275), anti-rabbit IgG HRP-linked (Cell Signaling Technology, catalog no. 7074, RRID: AB_2099233; 1:4000), and anti-mouse IgG HRP-linked (Cell Signaling Technology, catalog no. 7076, RRID: AB_330924; 1:4000).

### Reverse transcription quantitative polymerase chain reaction

RNAs were extracted from livers using the RNeasy Mini Kit (QIAGEN, catalog no. 74101) and quantified using a NanoDrop200c (Thermo Fisher Scientific). Complementary DNAs (cDNAs) were produced using the High-Capacity RNA-to-cDNA Kit (Thermo Fisher Scientific, catalog no. 4388950) according to the manufacturer’s instruction. Quantitative polymerase chain reactions (qPCRs) were performed using the DyNAmo HS SYBR Green qPCR Kit (Thermo Fisher Scientific, catalog no. F-410) on a Step-One Plus (Applied Biosystems) as follows: 20 s at 95°C, followed by 40 cycles of 3 s at 95°C, and 30 s at 60°C. mRNA quantification was calculated using ∆*C*_t_ method. The following mouse primers were used: mouse *Ctgf* (QIAGEN, QT00174020), mouse *Ctgf* (QIAGEN, QT00096131), mouse *Cyr61* (QIAGEN, QT00245217), mouse *Areg* (QIAGEN, QT00112217), 18*S* forward (5′-GTAACCCGTTGAACCCCATT-3′), and 18*S* reverse (5′-CCATCCAATCGGTAGTAGCG-3′).

### Quantification and statistical analysis

For IHC studies, five representative pictures were taken per mouse and were analyzed using Fiji software. For all in vivo studies, data are shown as means ± SD. Sample normality was assessed by Shapiro-Wilk test. Statistical significances were determined by two-tailed unpaired Student’s *t* test for two-group comparison, two-way analysis of variance (ANOVA) with Tukey or Dunnett for multiple group comparison, and log-rank (Mantel-Cox) test for survival comparison using GraphPad Prism software. Results were considered statistically different when *P < 0.05, **P < 0.01, ***P < 0.001, and ****P < 0.0001 with ns indicating no significance.

## SUPPLEMENTARY MATERIALS

Supplementary material for this article is available at http://advances.sciencemag.org/cgi/content/full/7/23/eabf9141/DC1

## Appendix

View/request a protocol for this paper from *Bio-protocol*.
